# Cost-Utility Analysis of COVID-19 Vaccination Strategies for Endemic SARS-CoV-2

**DOI:** 10.1001/jamanetworkopen.2025.15534

**Published:** 2025-06-13

**Authors:** Rafael N. Miranda, Alison E. Simmons, Michael W. Z. Li, Gebremedhin B. Gebretekle, Min Xi, Marina I. Salvadori, Bryna Warshawsky, Eva Wong, Raphael Ximenes, Melissa K. Andrew, Beate Sander, Davinder Singh, Sarah Wilson, Matthew Tunis, Ashleigh R. Tuite

**Affiliations:** 1 Centre for Immunization Surveillance and Programs, Public Health Agency of Canada, Ottawa, Ontario, Canada; 2 Dalla Lana School of Public Health, University of Toronto, Toronto, Ontario, Canada; 3 Public Health Risk Sciences Division, National Microbiology Laboratory, Public Health Agency of Canada, Guelph, Ontario, Canada; 4 Department of Mathematics and Statistics, McMaster University, Hamilton, Ontario, Canada; 5 Department of Pediatrics, McGill University, Montreal, Quebec, Canada; 6 Department of Epidemiology and Biostatistics, Western University, London, Ontario, Canada; 7 Department of Medicine (Geriatrics), Dalhousie University, Halifax, Nova Scotia, Canada; 8 Toronto General Hospital Research Institute, University Health Network, Toronto, Ontario, Canada; 9 Institute of Health Policy, Management and Evaluation, University of Toronto, Toronto, Ontario, Canada; 10 Public Health Ontario, Toronto, Ontario, Canada; 11 ICES, Toronto, Ontario, Canada; 12 Department of Community Health Sciences, Max Rady College of Medicine, University of Manitoba, Manitoba, Canada

## Abstract

**Importance:**

With shifting epidemiology and changes in the vaccine funding landscape, resource use considerations for COVID-19 vaccination programs are increasingly important.

**Objective:**

To assess the cost effectiveness of COVID-19 vaccination programs, where eligibility is defined by combinations of age and chronic medical conditions, including a strategy similar to current Canadian recommendations.

**Design, Setting, and Participants:**

Static, individual-based, probabilistic cost-utility model economic evaluation parameterized with recent data describing COVID-19 epidemiology, vaccine characteristics, and costs. The analysis used a 15-month time horizon from July 2024 to September 2025 and a modeled cohort of 1 million people with characteristics based on the Canadian population, stratified by age group and presence or absence of at least 1 chronic medical condition.

**Exposure:**

Annual or biannual COVID-19 vaccination strategies offered to different age and medical risk groups, with annual vaccination occurring in October and November in the primary analysis.

**Main Outcomes and Measures:**

Medically attended SARS-CoV-2 infections treated in outpatient and inpatient settings, including post-COVID condition cases and deaths. Costs in 2023 Canadian dollars, quality-adjusted life years (QALYs), and incremental cost-effectiveness ratios (ICERs), discounted at 1.5% for the health system and societal perspectives.

**Results:**

Among 1 million simulated individuals, annual vaccination for adults aged 65 years and older consistently emerged as a cost-effective intervention, with ICERs less than CAD $50 000 per QALY compared with no vaccination for a range of model assumptions. Adding a second dose for adults aged 65 years and older or expanding programs to include vaccination for younger age groups, including those at higher risk of COVID-19 due to chronic medical conditions, generally resulted in ICERs greater than $50 000 per QALY. Shifting timing of vaccination programs to better align with periods of high COVID-19 case occurrence resulted in biannual vaccination for those aged 65 years and older being cost effective.

**Conclusions and Relevance:**

In this economic evaluation of COVID-19 vaccination strategies, programs were observed to be cost effective when focused on groups at higher risk of disease. Optimal timing of programs improved the cost effectiveness of vaccination strategies. As COVID-19 transitioned to an endemic disease with high levels of population immunity, many jurisdictions revisited COVID-19 vaccination recommendations; these results identified COVID-19 vaccination programs that may provide good value for money.

## Key Points

**Question:**

What is the cost-effectiveness of different COVID-19 vaccination strategies?

**Findings:**

In this model-based economic evaluation of 1 million simulated individuals, annual vaccination of adults aged 65 years and older was estimated to be a cost-effective intervention, while the cost effectiveness of vaccination for younger ages and biannual vaccination for older adults was dependent on model assumptions.

**Meaning:**

These findings suggest COVID-19 vaccination programs may be cost effective when focused on those at higher risk of severe outcomes following infection.

## Teaser

This economic evaluation estimates the cost-effectiveness of COVID-19 vaccination programs in Canada.

## Introduction

Since the emergence of SARS-CoV-2 in 2019, COVID-19 has caused a high disease burden worldwide. By fall 2024, more than 7 million deaths had occurred globally.^1^ Although no longer a public health emergency of international concern,^2^ COVID-19 remains a significant cause of morbidity and mortality, particularly among higher risk groups, including older adults and immunocompromised individuals.^3,4,5^

The economic value of vaccination during the COVID-19 pandemic has been documented,^6,7,8,9^ and with waning immunity following infection and vaccination, as well as the emergence of new SARS-CoV-2 subvariants,^10^ the administration of additional vaccine doses remains an important tool for optimizing protection.^11^ Despite the effectiveness of vaccines for preventing severe COVID-19 outcomes, the cost effectiveness of broad vaccination programs has become less certain as SARS-CoV-2 is now circulating in populations with prior immune experience derived from infection and/or vaccination. The benefits of COVID-19 vaccination are not uniform; for instance, younger adults and children without comorbidities have a lower risk of severe outcomes and may benefit less.^12^

Before 2025, Canadian provinces and territories were accessing COVID-19 vaccines procured under federal pandemic investments, with vaccine costs assumed by the federal government. In 2025, provinces and territories transitioned to usual procurement pathways and now bear the cost of purchasing COVID-19 vaccines.^13^

Given the shifting epidemiology and changing vaccine funding landscape in Canada, there is an increased focus on efficient use of limited health care resources. The objective of this study was to estimate the cost-utility of age- and medical-risk based COVID-19 vaccination strategies in Canada.

## Methods

### Setting

Several COVID-19 vaccines are approved for use in Canada, where an expert advisory committee of the federal government provides recommendations for the use of vaccines but vaccination program decision-making and implementation falls within provincial and territorial responsibility. For fall 2024, Canada’s National Advisory Committee on Immunization (NACI) strongly recommended annual COVID-19 vaccination for people aged 65 years and older and people aged 6 months to 64 years who are at increased risk of SARS-CoV-2 infection or severe COVID-19 disease.^13^ For others aged 6 months to 64 years at lower risk of severe disease, NACI recommended a discretionary annual dose.^13^ NACI also has a discretionary recommendation for a second vaccine dose at a 6-month interval (with a minimum interval of 3 months) following the first dose for people aged 65 years and older, adult residents of long-term care homes and other congregate living settings for seniors, and people aged 6 months to 64 years who are moderately to severely immunocompromised.^14^

### Model Overview

We conducted a model-based cost-utility analysis of COVID-19 vaccination programs in Canada, estimating the cost effectiveness of vaccination strategies based on age and medical risk. The model timeframe was July 2024 to September 2025 (15 months). We used a discount rate of 1.5% for long-term costs and outcomes, with costs measured in 2023 Canadian dollars. Health outcomes were measured in quality-adjusted life years (QALYs). We assessed incremental cost-effectiveness ratios (ICERs) from both the health system (presented in the main text) and societal perspectives (eFigure 4, eTable 4, and eTable 5 in Supplement 1).^15^ The study followed Consolidated Health Economic Evaluation Reporting Standards (CHEERS) reporting guideline.^16^ No ethics review or approval was required or obtained for this model-based economic evaluation that used publicly-available data in accordance with 45 CFR §46.

### Model Structure

Our static, individual-based model of COVID-19 cases requiring medical care followed a closed population of 1 million people stratified by age group and medical risk status (eFigure 1 in Supplement 1) and used monthly time steps. It was adapted from a previously described model.^17^ The age group distribution and proportion of the population with 1 or more chronic medical conditions (CMCs) were based on data for the Canadian population.^18,19^ People without CMCs were considered average risk, and those with CMCs considered higher risk for experiencing severe outcomes following SARS-CoV-2 infection.

Medically attended COVID-19 was defined as requiring either outpatient (ie, health care practitioner or emergency department visit) or inpatient (ie, hospital admission, with or without intensive care unit admission) care. We assumed that a proportion of people with medically attended COVID-19 developed post-COVID condition (PCC)^20^ and that a proportion of people hospitalized with COVID-19 died.^21^ We assumed that individuals could have at most 1 medically attended illness episode attributable to COVID-19 during the modeled period.

Model parameters for COVID-19 epidemiology, vaccine characteristics, costs, health utilities, and data sources^7,13,18,20,21,22,23,24,25,26,27,28,29,30,31,32,33,34,35,36,37,38,39,40,41,42,43,44,45,46,47,48,49,50,51,52,53,54,55^ are described in more detail in Table 1 and Table 2 and in the eMethods in Supplement 1. When ranges are provided, this indicates parameter values were drawn from beta distributions for probabilities and utilities and from gamma distributions for costs.

**Table 1.** Population, SARS-CoV-2 Epidemiology, and Vaccine Characteristics Input Parameters

Parameter (source)	Base value (range)^a^
Population distribution by age, % (Statistics Canada^22^)	
<5 y	4.7
5-19 y	13.9
20-49 y	42.7
50-64 y	19.1
65-74 y	11.1
≥75 y	8.6
Population with 1 or more CMC by age, % (Queenan et al,^18^ 2021; Statistics Canada^23^; assumption)	
<5 y	14.4
5-19 y	21.0
20-49 y	24.9
50-64 y	45.6
65-74 y	60.1
≥75 y	72.1
Monthly distribution of annual COVID-19 cases: hospitalization data, % (Government of Canada^24^)	
July	3.7
August	3.7
September	12.5
October	15.2
November	16.0
December	19.0
January	10.4
February	6.1
March	4.7
April	3.0
May	3.7
June	1.9
Monthly distribution of annual COVID-19 cases, % (RVDSS^25^)	
July	5.1
August	8.4
September	12.4
October	13.4
November	13.2
December	12.4
January	8.7
February	5.5
March	4.2
April	3.7
May	5.7
June	7.4
Monthly distribution of annual COVID-19 cases: model projection, % (transmission model^26^)	
July	2.9
August	5.3
September	8.7
October	14.2
November	19.6
December	20.8
January	10.8
February	5.9
March	4.2
April	2.9
May	2.5
June	2.1
Annual incidence of medically-attended COVID-19 treated in an outpatient setting by age, % (transmission model^13^)	
0-4 y	10.6
5-19 y	7.5
20-49 y	7.5
50-64 y	10.6
≥65 y	12.0
Relative risk of medically-attended outpatient care in people with 1 or more CMCs compared with people without a CMC, % (assumption)	
All ages	1.5
Annual incidence of COVID-19-attributable hospitalization per 100 000 population by age (transmission model^13^)	
0-4 y	33.7
5-19 y	11.6
20-49 y	20.6
50-64 y	64.0
≥65 y	506.9
Patients hospitalized with confirmed COVID-19 with 1 or more CMCs by age, % (COVID-NET^27^)	
0-4 y	49.3
5-19 y	53.7
20-49 y	82.2
50-64 y	97.3
≥65 y	98.9
Patients hospitalized with COVID-19 who require ICU admission, % (CIHI,^28^ 2024)	
All ages	13.2 (12.7-13.6)
COVID-19 mortality per hospitalization by age, % (transmission model^26^; Centers for Disease Control and Prevention^21^; assumption)	
0-4 y	2.9
5-19 y	2.9
20-49 y	3.3
50-64 y	9.5
65-74 y	13.7
≥75 y	17.9
All-cause mortality rate per year, per 1000 population (Statistics Canada^29^)	
All ages	Age-specific rates
Outpatient COVID-19 duration, d (Centers for Disease Control and Prevention^21^; Government of Ontario^30^)	
All ages	5 (1-10)
Length of stay in hospital, d (CIHI,^28^ 2024)	
All ages	24 (16.2-48.6)
Length of stay in ICU, d (CIHI,^28^ 2024)	
All ages	9.4 (2.6-15.9)
Patients with medically attended COVID-19 prescribed an antiviral (Kuang et al,^31^ 2023)	
≥20 y	3.1 (2.5-3.7)^b^
Incremental vaccine effectiveness for COVID-19 infection requiring outpatient medical care, by time since vaccination, % (Link-Gelles^32^ 2024)	
First 2 mo	50
3 to 4 mo	32
5 to 6 mo	1
Incremental vaccine effectiveness for COVID-19 infection requiring hospitalization, by time since vaccination, % (Link-Gelles^32^ 2024)	
First 2 mo	49
3 to 4 mo	43
5 to 6 mo	14
Vaccine effectiveness weight for people with 1 or more CMC (assumption)	
All ages	0.8
Vaccination coverage: fall dose by age, % (Public Health Agency of Canada^33^)	
0-4 y	5.7
5-19 y	6.3
20-49 y	9.1
50-64 y	23.8
≥65 y	52.7
People receiving fall dose who receive a 6-mo booster, % (Public Health Agency of Canada^33^; assumption)	
All ages	20.9
Time to receive vaccine, including travel and waiting time, h (Canada Health Infoway^34^; Ray et al,^35^ 2015)	
All ages	2 (1.5-2.5)
Administration-based vaccine wastage rate, % (Office of the Auditor General of Ontario^36^; assumption)	
All ages	10
Adverse events following immunization, per 100 000 doses administered (Public Health Agency of Canada^37^)	
Serious adverse event	1.1
Duration of adverse event following immunization, d (Lee et al,^38^ 2009)	
Serious adverse event	2 (1-4)
Risk of developing PCC among outpatient cases by age, % (Prosser,^20^ 2024; assumption)	
0-4 y	0.5 (0.3-0.6)
5-19 y	0.7 (0.5-0.9)
≥20 y	2.3 (1.7-2.9)
Risk of developing PCC among hospitalized cases by age, % (Prosser,^20^ 2024; assumption)	
0-4 y	0.8 (0.3-1.3)
5-19 y	1.2 (0.5-2.0)
≥20 y	4.0 (1.7-6.4)
Duration of PCC, d (Statistics Canada^39^)	
All ages	365
PCC cases that miss school or work, %	16
Duration of school or work missed due to PCC	23.3

Abbreviations: CIHI, Canadian Institute for Health Information; CMC, chronic medical condition; ICU, intensive care unit; PCC, post-COVID-19 condition; RVDSS, Respiratory Virus Detection Surveillance System.

^a^ Ranges are included for parameters that were drawn from distributions.

^b^ Range defined as ±20% of the base value.

**Table 2.** Cost and Utility Input Parameters

Parameter (source)	Base value (range), CAD $^a^
Vaccination cost per dose	
Vaccine (Centers for Disease Control and Prevention^40^; assumption)	43 (43-107)
Administration (O’Reilly et al,^41^ 2017)	18 (13-22)
Transportation and storage (Sah et al,^7^ 2022)	0.64
Attributable medical costs per person with COVID-19 not requiring hospitalization (Sander et al,^42^ 2024)	
All ages	295 (207-383)
Attributable medical costs per person hospitalized with COVID-19 (Sander et al,^42^ 2024)	
Hospitalization without ICU admission	30 147 (28 760-31 534)
Hospitalization with ICU admission	105 677 (100 467-110 888)
Direct medical costs for severe adverse event following vaccination by age, y (Sander et al,^43^ 2010; CIHI^44^; Lee et al,^38^ 2009**)**	
<65	62 (48-82)
≥65	66 (51-87)
Transportation costs	
Cost of 2-way travel to vaccination or outpatient care (out of pocket) (Canada Health Infoway^34^)	14 (11-16)^b^
Cost of travel to inpatient care (NACI^45^)	417 (210-623)
Copayment per prescription (University of Western Ontario^46^; The Commonwealth Fund^47^; assumption)	
≥20 y	10 (5-12)
Out-of-pocket medication costs	
Over-the-counter medication costs for medically-attended case (Federici et al,^48^ 2018)	12 (3-20)
Prescription medication costs for severe adverse event following vaccination (<65 y) (Lee et al,^38^ 2009)	4.19 (3.14-5.24)
Antiviral costs (20-64 y) (CADTH,^49^ 2024)	1289 (1031-1547)^b^
Caregiver workdays lost, % (Keita Fakeye et al,^50^ 2023)	
Reduction in productivity	33
Labor force participation by age, % (Statistics Canada^51^)	
5-19 y	16.9
20-49 y	87.1
50-64 y	74.6
65-74 y	18.7
≥75 y	8.0
Caregiver	88.8
Average employment income by age (Statistics Canada^51^)	
5-19 y	6165
20-49 y	59 425
50-64 y	70 342
≥65 y	30 859
Caregiver (age 25-54)	69 095
Background health utility by age, mean preference weight	
0-4 y (Assumption)	0.950
5-19 y (Molina et al,^52^ 2023)	0.917
20-49 y (Molina et al,^52^ 2023)	0.880
50-64 y (Yan et al,^53^ 2023)	0.845
65-74 y (Yan et al,^53^ 2023)	0.867
≥75 y (Yan et al,^53^ 2023)	0.861
QALY loss for outpatient care by age, total (Prosser,^20^ 2024)	
0-4 y	0.00575 (0.00301-0.0085)
0-19 y	0.00561 (0.00287-0.00835)
≥20 y	0.0045 (0.0018-0.0074)
QALY loss for hospitalization without ICU admission by age, total (Prosser,^20^ 2024)	
0-19 y	0.01875 (0.0052-0.0324)
≥20 y	0.0174 (0.0038-0.031)
QALY loss for hospitalization with ICU admission by age, total (Prosser,^20^ 2024)	
0-19 y	0.08341 (0.0592-0.111)
≥20 y	0.0394 (0.0231-0.0583)
QALY loss for PCC following outpatient care by age, total	
0-4 y (Assumption)	0.1206 (0.0899-0.1568)
5-20 y (Prosser,^20^ 2024)	0.1126 (0.0830-0.1481)
≥20 y (Prosser,^20^ 2024)	0.0608 (0.038-0.0912)
QALY loss for PCC following infection treated in hospital by age, total	
0-4 y (Assumption)	0.1412 (0.1053-0.1836)
5-20 y (Prosser,^20^ 2024)	0.1319 (0.09719-0.1734)
≥20 y (Prosser,^20^ 2024)	0.0712 (0.0445-0.1068)
QALY loss for death by age, total (Statistics Canada^29,54^)	
0-4 y	41.3844
5-19 y	38.1545
20-49 y	29.5843
50-64 y	19.0662
65-74 y	13.1362
≥75 y	7.3480
Quality-adjusted life days lost for adverse event following vaccination, total (Sandmann et al,^55^ 2021)	
Serious adverse event	1.0 (0.75-1.25)

Abbreviations: CADTH, Canadian Agency for Drugs and Technologies in Health; CIHI, Canadian Institute for Health Information; ICU, intensive care unit; PCC, post-COVID-19 condition; QALY, quality-adjusted life-year.

To convert CAD $ to US $, multiply by 0.73.

^a^ Ranges are included for parameters that were drawn from distributions. Range defined as ±20% of the base value.

### COVID-19 Epidemiology and Disease History

We used an age-stratified dynamic transmission model that incorporated immunity levels, calibrated to COVID-19 hospital occupancy (January 2022 to April 2024) and seroprevalence data (January 2022 to December 2023),^26^ to project annual cumulative incidence of symptomatic and hospitalized COVID-19 cases in the absence of any COVID-19 vaccination occurring from July 2024 to September 2025 (no vaccination counterfactual). COVID-19 cases were assumed to follow the monthly distribution of hospitalized cases reported from July 2023 to June 2024.^24^

### Utilities

We used age-specific utilities based on EQ-5D-5L index scores to calculate QALY losses from COVID-19 mortality.^52,53^ QALY losses from other health outcomes were based on publicly-available data^20^ and assumptions.

### Costs

We used health care costs attributable to COVID-19 for a 1-year period following initial diagnosis for people treated in either outpatient or inpatient settings.^42^ Post-acute care costs were assumed to include costs associated with PCC. In the absence of Canadian list prices for COVID-19 mRNA vaccines, we used a price of $43 per dose for our base case, based on an unpublished Public Health Agency of Canada analysis of the historic relation between Canadian negotiated vaccine prices and US Centers for Disease Control and Prevention (CDC) public list prices (to convert CAD $ to US $, multiply by 0.73).^40^ We also included costs for vaccine administration, adverse events following immunization, and doses procured but not administered. Societal perspective costs included patient productivity loss due to COVID-19 disease and death, vaccination and adverse events following immunization, caregiver productivity loss, and out-of-pocket medical costs. Productivity loss was estimated using the human capital and friction cost methods.^15^

### Vaccination

Vaccination was assumed to occur over 2 months (October and November for the base case), with a second dose 6 months after the first (for those receiving it). Vaccine coverage was based on estimates of uptake in the spring 2023 and fall and winter 2023 to 2024 vaccination campaigns.^33^ We assumed different vaccine effectiveness (VE) and waning values for the outcomes of medically attended outpatient and inpatient cases and lower VE for people at higher risk of COVID-19 disease than those at average risk (eFigure 2 in Supplement 1).^32^

### Vaccination Strategies

In addition to the no vaccination strategy (no vaccination beyond those received before July 2024), we evaluated a series of increasingly inclusive annual vaccination strategies: all aged 65 years and older; all aged 65 years and older and higher risk individuals aged 50 to 64 years; all aged 65 years and older and higher risk individuals aged less than 65 years; and all aged 50 years and older and higher risk individuals aged less than 50 years. Each strategy was evaluated with and without a second dose (ie, biannual vaccination) for those aged 65 years and older. The strategy of biannual vaccination for people aged 65 years and older and annual vaccination for higher risk people aged less than 65 years had features most similar to NACI recommendations at the time of the analysis.

### Statistical Analysis

We conducted 2000 model simulations per strategy. We calculated mean QALYs and costs and conducted a sequential analysis to compare ICERs, eliminating dominated or extended dominated strategies.^56^ We also calculated outcomes averted compared with no vaccination and number needed to vaccinate, presented as medians and 95% credible intervals (CRI).

We conducted a probabilistic sensitivity analysis for the base-case analysis. We performed a sensitivity analysis for vaccine price by identifying the strategy with the largest sequential ICER below the specified cost-effectiveness threshold at different vaccine prices. We also conducted scenario analyses focusing on estimates of vaccine wastage, vaccine prices, COVID-19 incidence, monthly distribution of COVID-19 cases (eFigure 3 in Supplement 1), and vaccination program timing relative to peak disease activity. Details of the scenarios are provided in eTable 1 in Supplement 1. All analyses were conducted using R version 4.2.2 (R Project for Statistical Computing).^57^

## Results

### Estimated Impact of Vaccination Strategies

From July 2024 to September 2025, we estimated 8665 (95% CRI, 8616-8713) outpatient cases, 133 (95% CRI, 127-140) inpatient cases, 178 (95% CRI, 138-225) PCC cases, and 16 (95% CRI, 14-18) deaths per 100 000 person-years for the no vaccination strategy (eTable 2 in Supplement 1).

**Figure 1.**
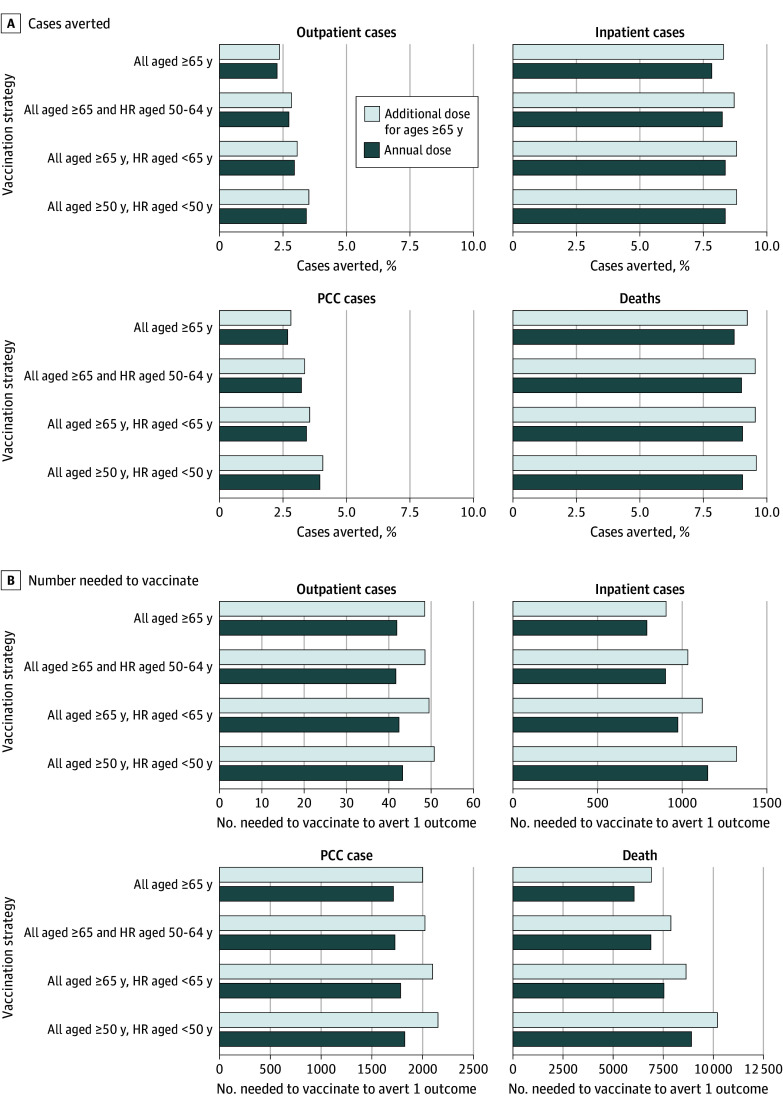
Estimated Impact of Vaccination on COVID-19 Health Outcomes A, Cases averted for different vaccination strategies compared with no vaccination based on COVID-19 coverage data for spring 2023 and fall 2023 to 2024. B, Number needed to vaccinate to avert COVID-19 health outcomes for different vaccination strategies. Darker bars denote annual vaccination for the indicated groups while lighter bars denote a second vaccine dose (at a 6-month interval) offered to the population aged 65 years and older and annual vaccination for all other specified groups. Note different x-axis scales for number needed to vaccinate estimates depending on the outcome assessed. HR indicates higher risk (1 or more chronic medical conditions).

Compared with no vaccination, vaccination had a larger proportional estimated impact on preventing COVID-19 hospitalizations and deaths than outpatient and PCC cases (Figure 1; eTable 2 in Supplement 1). The estimated proportion of outcomes averted increased as program eligibility increased. Compared with no vaccination for the population, we estimated that 2.3% to 3.5% of outpatient cases, 7.8% to 8.8% of inpatient cases, 2.7% to 4.1% of PCC cases, and 8.7% to 9.6% of deaths could be averted for the vaccination strategies considered based on recent coverage estimates for 2023 to 2024. The number needed to vaccinate increased for the prevention of each inpatient case, PCC case, or death as program eligibility was expanded to include younger ages. The number needed to vaccinate to prevent an outpatient case remained low even when younger ages were included. Number needed to vaccinate increased when a second dose for adults 65 years and older was included compared with only annual vaccination for this age group.

### Base-Case Cost-Effectiveness Analysis

**Figure 2.**
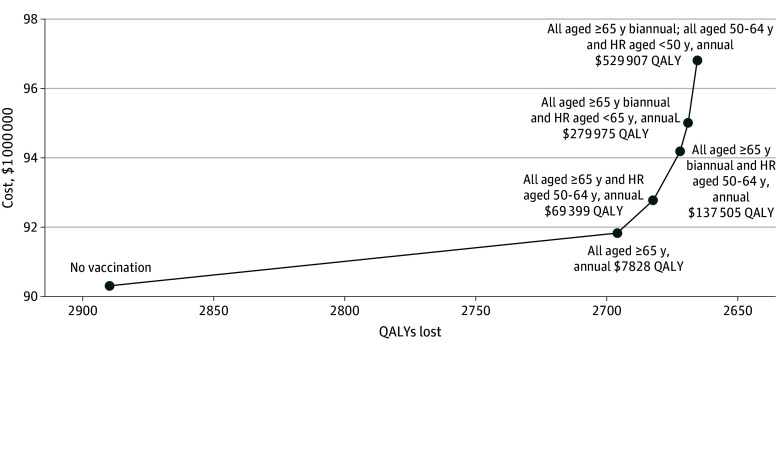
COVID-19 Vaccination Strategy Costs, Quality-Adjusted Life Year (QALY) Losses, and Incremental Cost-Effectiveness Ratios (ICERs) Dominated or extended dominated strategies are excluded. The solid line shows the cost-effectiveness frontier that connects nondominated strategies. Labels show the strategies and sequential ICERs for each strategy, with the ICER calculated relative to the prior (less costly) strategy on the frontier. Results are shown for the base-case analysis for the health system perspective. HR indicates higher risk (1 or more chronic medical conditions).

In the base-case analysis, we found that compared with no vaccination beyond those doses received in the past, annual vaccination for all adults aged 65 years and older resulted in an ICER of $7828 per QALY (Figure 2; eTables 3, 4, and 5 in Supplement 1). Expansion to include an annual dose for higher-risk adults aged 50 to 64 years resulted in an ICER of $69 399 per QALY compared with an annual dose for adults aged 65 years and older. The next most cost-effective strategy was the addition of a second dose for the 65 years and older age group, with an ICER of $137 505 per QALY compared with annual vaccination for those aged 50 to 64 years at higher risk and all those 65 years and older. Finally, expansion to include annual vaccination for the population at higher risk aged less than 50 years resulted in an ICER of $279 975 per QALY compared with a biannual dose for adults aged 65 years and older and an annual dose for higher-risk adults aged 50 to 64 years. Adding the population at average risk aged 50 to 64 years (for whom there is currently only a discretionary, not a strong, recommendation) to receive annual COVID-19 vaccination resulted in an ICER of $529 907 per QALY compared with the current recommendation of biannual vaccination for those aged 65 years and older and annual vaccination for those at higher risk aged less than 65 years.

Probabilistic sensitivity analysis showed that at a $50 000 per QALY cost-effectiveness threshold, annual vaccination for adults aged 65 years and older had the largest probability of being cost effective (eFigure 4 in Supplement 1). Using a cost-effectiveness threshold of $50 000 per QALY, we found that annual vaccination for all adults aged 65 years and older remained the optimal strategy when vaccine price was between $32 and $110 per dose (eFigure 5 in Supplement 1). Vaccine price would need to be between $12 and $31 per dose for annual vaccination of all adults 65 years and older and higher-risk adults aged 50 to 64 years to be the optimal strategy. A vaccine price less than $12 per dose was required for biannual vaccination for all adults 65 years and older plus annual vaccination for higher-risk adults 50 to 64 years to be the optimal strategy.

### Scenario Analyses

The ICER for annual vaccination for adults aged 65 years and older compared with no vaccination remained less than $50 000 per QALY across all scenarios considered, including if vaccine price was increased from $43 per dose included in the base case to $107 per dose (Table 3). By contrast to the results for the strategy of annual vaccination for adults aged 65 years and older, and consistent with the base-case analysis, all other vaccination strategies resulted in sequential ICERs greater than $50 000 per QALY in scenario analyses, with 3 exceptions. First, if COVID-19 incidence had alternate seasonality with a flatter curve, such that infection was less concentrated in the winter months and a higher proportion of cases occurred in the spring and summer months than assumed in the base-case analysis, offering a second vaccine dose for adults aged 65 years and older resulted in an ICER of $44 278 per QALY compared with annual vaccination for this age group. Second, for a scenario assuming an earlier program start and shorter (4-month) interval between doses for those receiving 2 doses, such that vaccine protection was highest for the 8 contiguous months of highest disease activity, adding a second dose for the population aged 65 years and older resulted in an ICER of $49 418 per QALY compared with annual vaccination for this age group. Finally, if VE was equivalent in high risk and average risk populations, the ICER for annual vaccination of all adults aged 65 years and older and higher-risk adults aged 50 to 64 years was $49 821 compared with annual vaccination for all adults aged 65 years and older.

**Table 3.** Sequential Incremental Cost-Effectiveness Ratios (ICERs) for All Vaccination Strategies Across Various Scenario Analyses

Strategy	Change from previous strategy	Sequential ICER (CAD $ per QALY)										
		Base case	Higher vaccine price		Higher vaccine wastage		Equal VE for HR and AR groups	Higher COVID-19 incidence	Alternate seasonality		Earlier program start	Earlier program start and 4-mo interval between doses
			$80 per dose	$107 per dose	20%	30%			Flatter curve	Larger winter wave		
≥65 y all annual (1)^a^	Annual dose for ≥65 y	7828	29 471	45 264	10 115	12 401	1154	7979	17 460	4923	7116	7024
≥65 y all and 50-64 y HR annual (2)	Add annual dose for 50-64 y HR (to 1)	69 399	130 250	174 656	75 828	82 257	49 821	67 469	D	59 860	69 918	D
65 y all biannual (3)	Add 6-mo dose for all ≥65 y (to 1)	D	D	D	D	D	D	D	44 278	D	D	49 418
≥65 y all biannual and 50-64 y HR annual (4)	Add 6-mo dose for 65 y all (to 2) or 50-64 y HR annual (to 3)	137 505	239 892	314 607	148 322	159 139	103 998	120 218	118 299	188 006	134 136	88 910
≥65 y all biannual and <65 y HR annual (5)	Add <50 y HR annual (to 4)	279 975	481 131	627 921	301 227	322 480	208 259	271 466	352 241	250 083	282 375	256 887
≥65 y all biannual, 50-64 y all and <50 y HR annual	Add 50-64 y AR (to 5)	529 907	885 301	1 144 643	567 455	605 002	552 549	548 001	718 679	505 301	589 384	576 314
≥65 y all and<65 y HR annual	NA	ED	ED	ED	ED	ED	ED	ED	D	ED	ED	D
≥50 y all and <50 y HR annual	NA	D	D	D	D	D	D	D	D	ED	D	D

Abbreviations: AR, average risk (no chronic medical conditions); D, dominated; ED, extended dominated; HR, higher risk (1 or more chronic medical conditions); NA, not applicable; QALY, quality-adjusted life-year; VE, vaccine effectiveness.

^a^ Comparator for this strategy is no vaccination.

## Discussion

Our economic evaluation of COVID-19 vaccination programs in Canada found that annual vaccination for the population aged 65 years and older was cost effective, even under more pessimistic assumptions. The next most efficient strategy tended to be program expansion to include adults aged 50 to 64 years with 1 or more chronic medical conditions, but ICERs generally exceeded the commonly used $50 000 per QALY threshold.

Results were sensitive to assumptions about program timing. Biannual vaccination for the population aged 65 and older was the optimal strategy in scenarios assuming a lower concentration of cases in the winter months or altered program timing to better align with periods of high COVID-19 activity. This likely reflects the high burden of severe COVID-19 in this age group, waning of vaccine protection over approximately a 6-month period, and the benefits of a second dose for preventing disease when vaccination protection is better aligned with periods of high COVID-19 disease incidence.

Economic evaluations to inform recommendations from other National Immunization Technical Advisory Groups similarly found COVID-19 vaccination for those at higher risk of severe disease was most cost effective.^58,59^ Economic evaluations reviewed by the UK Joint Committee on Vaccination and Immunisation identified a biannual program with universal vaccination for older adults, residents of care homes for older adults, and immunosuppressed individuals aged 6 months and older as cost effective.^58^ The optimal age cutoff for older adults was sensitive to vaccine price, which was unknown at the time of the analysis. For a combined vaccine price and delivery cost of $44, vaccination for adults 75 years and older was optimal for a cost-effectiveness threshold of $35 226 (£20,000) per QALY, but age cutoffs of 70 years or 80 years were optimal for lower or higher vaccine prices, respectively. An economic analysis reviewed by the American Committee on Immunization Practices estimated an ICER of CAD $80 443 CAD per QALY for annual vaccination of adults aged 65 years and older compared with no vaccination using a vaccine price of $182.^59^ ICERs for a second dose for this age group or for an annual dose for those aged 5 to 64 years exceeded $280 000 per QALY. In a sensitivity analysis using a vaccine price of $41 per dose (similar to our value of $43), vaccinating adults aged 65 years and older with 1 annual dose was less costly and more effective than a strategy of no vaccination, while biannual vaccination for this age group exceeded $115 000 per QALY compared with annual vaccination.

### Limitations

Although we conducted scenario analyses to address uncertainty associated with model assumptions, our analysis had limitations. To account for the effects of ongoing SARS-CoV-2 exposure and the resulting accumulation and waning of population immunity on COVID-19 incidence, we used a dynamic transmission model to estimate the no-vaccination counterfactual but used a static model to estimate vaccination impact. This allowed for greater flexibility regarding assumptions about vaccine effectiveness, including differences in magnitude and duration of protection for different outcomes. This approach excluded indirect benefits of vaccination, but given low assumed vaccine coverage (based on data from previous years), the benefits of vaccination are likely primarily direct. The focus on medically attended cases underestimates the societal impacts of infections not requiring medical care.^60^ Similarly, we are not capturing productivity in the informal labor market, resulting in conservative estimates of the societal benefits for vaccination for people aged 65 and older.^60^ In the absence of Canadian vaccine price data, we used US CDC public list prices for mRNA vaccines and historical data on relative price differentials between CDC public list prices and Canadian negotiated vaccine prices. We used vaccine price for mRNA vaccines, since they represented the majority of vaccines currently used in Canada. Applying this discount to the Nuvaxovid protein subunit vaccine^40^ yields a vaccine price of $34 per dose, close to the price at which program expansion to include higher risk individuals aged 50 to 64 years resulted in ICERs under $50 000 per QALY. Additionally, our analysis only included key population groups included in current Canadian recommendations and did not include all those aged less than 65 years who are currently strongly recommended annual vaccination or who are recommended to receive biannual vaccination based on a NACI discretionary recommendation.^13,14^ We also grouped all adults aged 65 and older together, consistent with recommendations at the time of the analysis; however, since severe illness risk increases with age, using higher age thresholds for vaccination could improve cost effectiveness, but would prevent fewer cases of severe illness.

## Conclusions

In this economic evaluation of COVID-19 vaccination strategies, COVID-19 vaccination programs in Canada remained cost effective when focused on populations at higher risk of COVID-19, despite changing epidemiology. Alignment of vaccine administration with periods of increased SARS-CoV-2 transmission increased the efficiency of vaccination programs.

## Notes

Supplement 1.
eMethods.
eFigure 1. **Model Health States and Transitions Between States** eFigure 2. **Incremental Vaccine Effectiveness (VE) Assumptions Used in the Cost-Effectiveness Analysis** eFigure 3. **Assumed Annual Distributions of COVID-19 Cases Used in the Cost-Effectiveness Analysis** eFigure 4. **Cost-Effectiveness Acceptability Curve for the Base-Case Probabilistic Sensitivity Analysis** eFigure 5. **Optimal Vaccination Strategy for a Range of Vaccine Price and Cost-Effectiveness Thresholds** **eTable 1.** Summary of Assumptions Used in Base-Case and Scenario Analyses **eTable 2.** Health Outcomes by Vaccination Strategy, Median and 95% Credible Interval **eTable 3.** Costs, QALYs, and Sequential ICERs for Base-Case Scenario by Strategy: Health System Perspective **eTable 4.** Costs, QALYs, and Sequential ICERs for Base-Case Scenario by Strategy: Societal Perspective (Human Capital Method) **eTable 5.** Costs, QALYs, and Sequential ICERs for Base-Case Scenario by Strategy: Societal Perspective (Friction Cost Method)
eReferences.

Supplement 2.
Data Sharing Statement
