# Supplementary material for: Cost-Utility Analysis of COVID-19 Vaccination Strategies for Endemic SARS-CoV-2
Source: JAMA Netw Open. 2025 Jun 13;8(6):e2515534. doi: 10.1001/jamanetworkopen.2025.15534 (PMC12166483; doi:10.1001/jamanetworkopen.2025.15534)
Supplement: Supplement 1. — eMethods. eFigure 1. Model Health States and Transitions Between States eFigure 2. Incremental Vaccine Effectiveness (VE) Assumptions Used in the Cost-Effectiveness Analysis eFigure 3. Assumed Annual Distributions of COVID-19 Cases Used in the Cost-Effectiveness Analysis eFigure 4. Cost-Effectiveness Acceptability Curve for the Base-Case Probabilistic Sensitivity Analysis eFigure 5. Optimal Vaccination Strategy for a Range of Vaccine Price and Cost-Effectiveness Thresholds eTable 1. Summary of Assumptions Used in Base-Case and Scenario Analyses eTable 2. Health Outcomes by Vaccination Strategy, Median and 95% Credible Interval eTable 3. Costs, QALYs, and Sequential ICERs for Base-Case Scenario by Strategy: Health System Perspective eTable 4. Costs, QALYs, and Sequential ICERs for Base-Case Scenario by Strategy: Societal Perspective (Human Capital Method) eTable 5. Costs, QALYs, and Sequential ICERs for Base-Case Scenario by Strategy: Societal Perspective (Friction Cost Method) eReferences. [file jamanetwopen-e2515534-s001.pdf]

Supplemental Online Content

Miranda RN, Simmons AE, Li MWZ, et al. Cost-utility analysis of COVID-19 vaccination strategies for endemic SARS-CoV-2. *JAMA Netw Open*. 2025;8(6):e2515534. doi:10.1001/jamanetworkopen.2025.15534

eMethods.

eFigure 1. Model Health States and Transitions Between States

eFigure 2. Incremental Vaccine Effectiveness (VE) Assumptions Used in the Cost-Effectiveness Analysis

eFigure 3. Assumed Annual Distributions of COVID-19 Cases Used in the Cost-Effectiveness Analysis

eFigure 4. Cost-Effectiveness Acceptability Curve for the Base-Case Probabilistic Sensitivity Analysis

eFigure 5. Optimal Vaccination Strategy for a Range of Vaccine Price and Cost-Effectiveness Thresholds

eTable 1. Summary of Assumptions Used in Base-Case and Scenario Analyses

eTable 2. Health Outcomes by Vaccination Strategy, Median and 95% Credible Interval

eTable 3. Costs, QALYs, and Sequential ICERs for Base-Case Scenario by Strategy: Health System Perspective

eTable 4. Costs, QALYs, and Sequential ICERs for Base-Case Scenario by Strategy: Societal Perspective (Human Capital Method)

eTable 5. Costs, QALYs, and Sequential ICERs for Base-Case Scenario by Strategy: Societal Perspective (Friction Cost Method)

eReferences.

This supplemental material has been provided by the authors to give readers additional information about their work.

© 2025 Miranda RN et al. *JAMA Network Open*.

eMethods

Model structure

Our static individual-based model of COVID-19 cases requiring medical care followed a closed population of 1 million people stratified by age group and medical-risk status (**eFigure 1**). It was adapted from a previously described model.[1] The age group distribution was based on projections for the Canadian population.[2] The presence of one or more chronic medical conditions (CMCs) was used to characterize the proportion of the population at higher risk (HR) for experiencing severe outcomes following SARS-CoV-2 infection.[3,4] The remaining proportion of the population without CMCs was characterized as average risk (AR). The model used monthly time steps.

Model parameters for COVID-19 epidemiology, vaccine characteristics, costs, and health utilities are described in more detail in **Table 1** and **Table 2**. We obtained parameters from published studies and available data, using Canadian sources when possible, and made assumptions if data were not available. When ranges are provided, this indicates parameter values drawn from distributions; beta distributions were used for probabilities and utilities, and gamma distributions were used for costs.

COVID-19 epidemiology and disease history

We used a separate age-stratified dynamic compartmental transmission model calibrated to COVID-19 hospital occupancy (from January 2022 to April 2024) and seroprevalence data (from January 2022 to December 2023) that includes vaccination within this fitting window and incorporates immunity levels.[5] We then estimated annual cumulative incidence of symptomatic and hospitalized COVID-19 cases in the absence of any COVID-19 vaccination between July 2024 and September 2025. This provided our "no vaccination" counterfactual. Details of the transmission model are provided elsewhere.[5] Annualized incidence estimates for the no vaccination counterfactual scenario were then used to estimate the impact of vaccination for preventing medically-attended COVID-19 cases using a static cost-effectiveness model. The use of a static model for estimating vaccination impact allowed for greater flexibility for evaluating different magnitudes and durations of vaccine protection associated with different health outcomes (i.e., medically attended COVID-19 managed in the outpatient or inpatient setting). This combined modelling approach allowed for the incorporation of population immunity due to ongoing infection and previous vaccination over the modelled time period.

The proportion of annual COVID-19 cases occurring each month was assumed to follow the monthly distribution of hospitalized cases reported from July 2023 to June 2024.[6] We estimated costs and QALYs associated with medically attended COVID-19 only, which was defined as requiring one of the following levels of care: outpatient (e.g., health care provider or emergency department visit) or inpatient (e.g., hospital admission, with or without intensive care unit (ICU) admission). We assumed that a proportion of people with medically attended COVID-19 developed post-COVID condition (PCC), with a higher risk among people who were hospitalized.[7] We also included costs and QALY losses for people with COVID-19 attributable mortality.

Utilities

We used age-specific utilities based on EQ-5D-5L index scores for the Canadian population to calculate QALY losses associated with COVID-19 mortality.[8,9] QALY losses associated with other modelled health outcomes were derived from published studies[7] and assumptions.

Costs

© 2025 Miranda RN et al. *JAMA Network Open*.

We used long-term health care costs attributable to diagnosed COVID-19 derived from a population-based matched cohort study in Ontario, Canada.[10] These cost estimates covered a period of approximately one year following an initial diagnosis between January and December 2020 for people treated in either outpatient or inpatient settings and included both acute care and post-acute care costs. Post-acute care costs were assumed to include costs associated with PCC and consequently, additional health care costs for PCC were not included in our analysis.

In the absence of Canadian list prices for COVID-19 vaccines, we used a price of $43 per dose for our base case. This estimate is 40% of the US Centers for Disease Control and Prevention (CDC) public list price of $107 per dose and was based on an unpublished Public Health Agency of Canada analysis of historic data that suggests that Canadian negotiated vaccine prices across all vaccine-preventable diseases are typically 30-50% of US public list prices. We explored vaccine prices of 75% ($80) and 100% ($107) of the CDC list price in scenario analyses. We also included costs associated with vaccine administration, adverse events following immunization (AEFIs), and vaccine doses procured but not administered. To account for vaccine wastage, we applied an administration-based wastage rate of 10% in the base case; in other words, for every 100 vaccine doses administered, an additional 10 doses were procured but not administered. The administration-based wastage rate was calculated as a percentage increase over the number of administered doses, to account for losses. This corresponds to a traditional wastage rate of approximately 9% when wastage is calculated as percentage of total doses procured. The administration-based approach simplifies calculations in our analysis, given that administered doses rather than procured doses are a model input. Costs for wasted doses included vaccine costs and transportation and storage costs and excluded administration costs. Costs for the societal perspective included patient productivity loss due to COVID-attributable disease and death, vaccination and AEFIs, caregiver productivity loss, and out-of-pocket medical costs. Productivity loss was estimated using the human capital and friction cost methods, using age-specific labour force participation rates and average employment income, as described previously.[1]

Vaccination

Vaccination was assumed to occur over a two-month period. In the base-case analysis, vaccination occurred in October and November and, for those receiving it, a second dose was administered 6 months after receipt of the first dose. Timing of dose administration was varied in scenario analyses. Vaccine coverage was based on Canadian estimates of uptake in the spring 2023 and fall/winter 2023-2024 vaccination campaigns.

We assumed different vaccine effectiveness (VE) and waning values for the outcomes of medically attended outpatient and inpatient cases and lower VE for people at higher risk of COVID-19 disease than those at average risk (**eFigure 2**). VE was assumed to decline over time, with protection falling to 1% against medically-attended outpatient COVID-19 and to less than 15% for COVID-19 requiring inpatient care in months 5 and 6 following vaccination. Protection against all outcomes was 0% by month 7 following vaccination. VE estimates were based on US observational data for the 2023-2024 season.[11] Vaccination was assumed to reduce risk of PCC by reducing overall SARS-CoV-2 infection risk; we did not include additional VE for preventing PCC in those who did become infected.

Vaccination strategies

Although COVID-19 vaccines are authorized for the population aged 6 months and older, the youngest modelled age group included the population aged 0 to 4 years, and we did not

© 2025 Miranda RN et al. *JAMA Network Open*.

model births in the population. For this reason, vaccination strategies that included a population aged under 5 years were assumed to apply to the entire age group.

We evaluated a series of increasingly inclusive annual vaccination strategies that added younger age groups, as follows:

- No vaccination beyond the vaccinations that had been received prior to July 2024
- All aged 65 years and older
- All aged 65 years and older and HR aged 50 to 64 years
- All aged 65 years and older and HR aged less than 65 years
- All aged 50 years and older and HR aged less than 50 years

Each of the four vaccination strategies was evaluated including and excluding a second dose (i.e. biannual vaccination) for the population aged 65 years and older (for a total of eight vaccination strategies in addition to the no vaccination strategy). This approach allowed us to examine the key components of the current strategy based on the incremental costs and effects associated with vaccinating the different population groups and the number of doses for those 65 years of age and older. The strategy of biannual vaccination for people aged 65 years and older and annual vaccination for higher risk people aged less than 65 years had features most similar to current NACI recommendations. The final strategy (all people 50 years of age and older along with vaccination for people younger than 50 years at higher risk), was more inclusive than the NACI guidance for those strongly recommended an annual dose at the time of the anlysis.

Analysis

For each vaccination strategy, we calculated QALYs and costs associated with the modelled health outcomes. We conducted a sequential analysis to compare ICERs. In a sequential analysis, a strategy is removed if others result in more QALYs gained at lower costs (the eliminated strategy is dominated) or if a strategy would never be the optimal intervention regardless of the cost-effectiveness threshold used (i.e., the eliminated strategy is subject to extended dominance).[12] ICER estimates represent the mean of 2,000 model simulations, with each simulation based on a unique draw from parameter distributions. The number of simulations was selected to ensure adequate sampling from probability distributions. We also calculated outcomes averted compared to no vaccination and number needed to vaccinate to avert an outpatient case, inpatient case, PCC case, or death. Summary results for health outcomes across strategies represent medians and 95% credible intervals (CrI) from the 2,000 model simulations.

Sensitivity and scenario analyses

We conducted a probabilistic sensitivity analysis for the base-case analysis to estimate the probability that competing vaccination strategies were cost-effective at varying cost-effectiveness thresholds. We performed a sensitivity analysis to explore the sensitivity of the base-case results to assumed vaccine price. For this analysis we identified the optimal strategy at different vaccine prices, with the optimal strategy identified as that with the largest sequential ICER that was below the specified cost-effectiveness threshold. We also conducted a range of scenario analyses focusing on estimates of administration-based vaccine wastage, specific vaccine prices, COVID-19 incidence, monthly distribution of COVID-19 cases (**eFigure 3**), and vaccination program timing relative to peak disease activity. Details of the scenarios are provided in **eTable 1**.

© 2025 Miranda RN et al. *JAMA Network Open*.

eFigure 1. Model Health States and Transitions Between States. Boxes indicate health states included in the model and arrows indicate possible transitions between health states. Transitions from any of the health states to death due to background mortality were also included in the model but arrows are not shown. Risk of experiencing medically attended COVID-19 health outcomes were dependent on age, medical risk status, and vaccination status. The model uses monthly time steps.

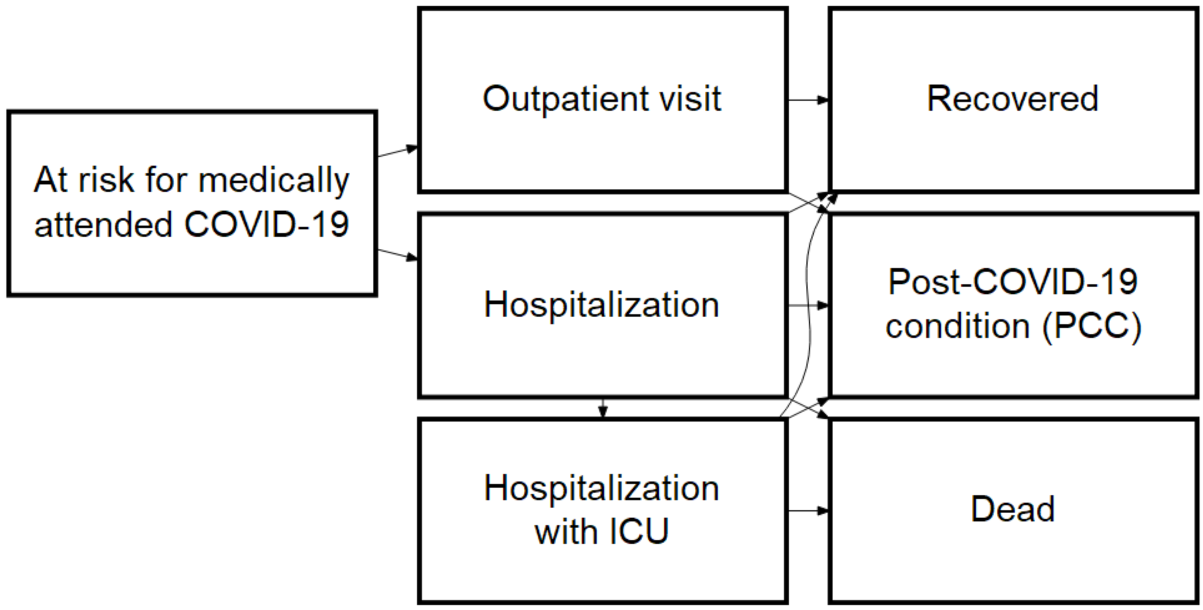

© 2025 Miranda RN et al. *JAMA Network Open*.

eFigure 2. Incremental Vaccine Effectiveness (VE) Assumptions Used in the Cost-Effectiveness Analysis. Different VE was assumed for protection against medically attended COVID-19 requiring outpatient or inpatient care. VE was assumed to be lower for people with chronic medical conditions placing them at higher risk of COVID-19 than for those at average risk in the base-case analysis. Incremental VE was assumed to wane over time, falling to 0 by seven months following vaccination. Note that outpatient VE is assumed to be 1% for months 5 and 6 following vaccination. For people receiving two COVID-19 vaccines a year, receipt of the second dose was assumed to return VE to initial values, with similar waning over time. The graph below assumes a six-month interval between doses, as was assumed in the base case analysis.

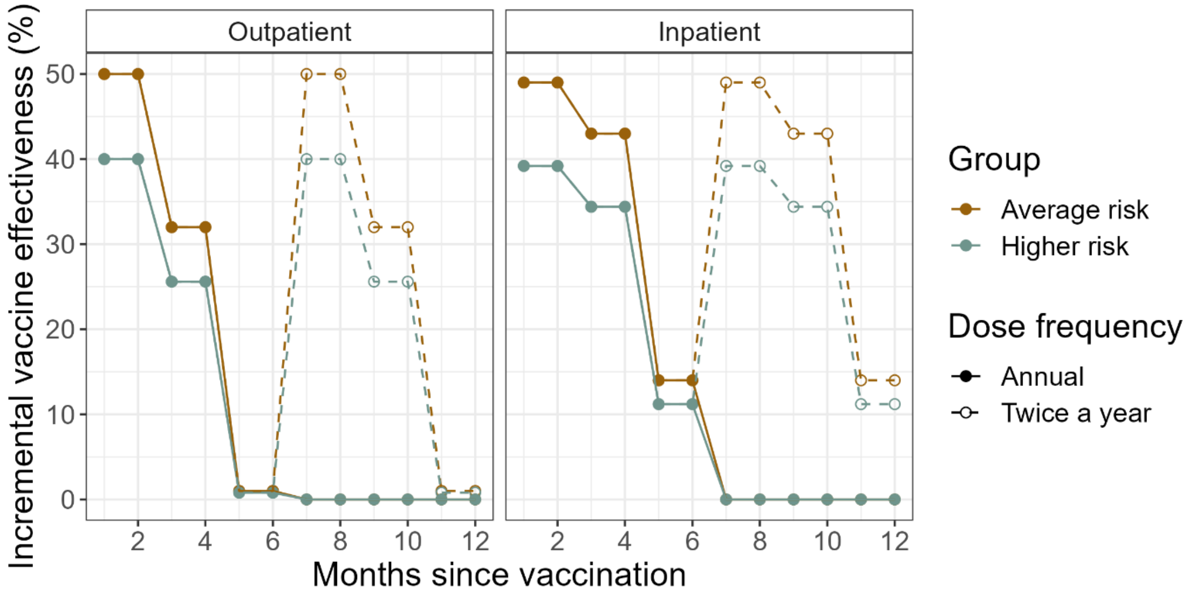

© 2025 Miranda RN et al. *JAMA Network Open*.

eFigure 3. Assumed Annual Distributions of COVID-19 Cases Used in the Cost-Effectiveness Analysis. The percent of annual COVID-19 cases occurring each month was assumed to follow hospitalization data in the base case analysis. Alternate monthly distributions obtained from the Respiratory Virus Detection Surveillance System (RVDSS) and a transmission model were used in scenario analyses. Hospitalization and RVDSS estimates are based on data for July 2023 to June 2024. The model estimates are based on a projection period covering July 2024 to June 2025.

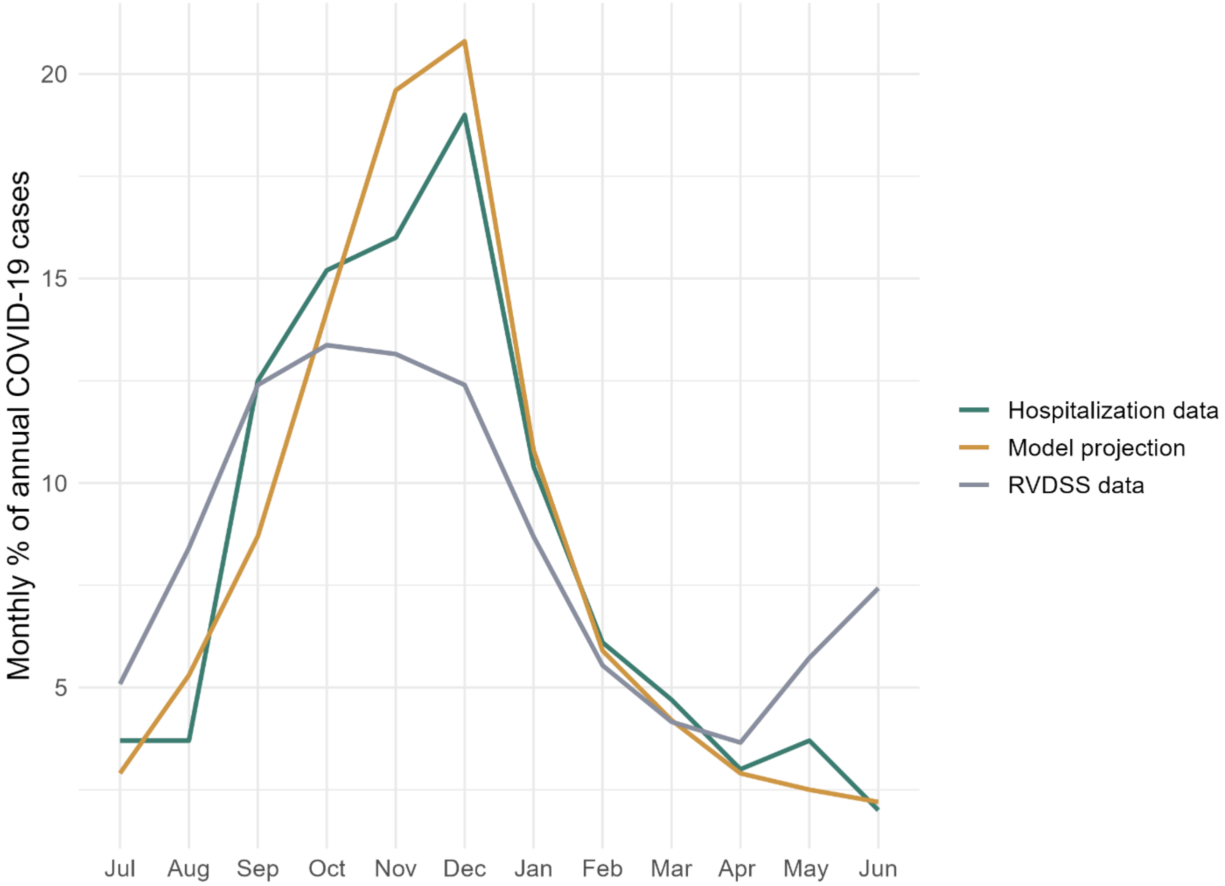

© 2025 Miranda RN et al. *JAMA Network Open*.

eFigure 4. Cost-Effectiveness Acceptability Curve for the Base-Case Probabilistic Sensitivity Analysis. Points show the proportion of samples for which a given vaccination strategy was identified as cost-effective using a given cost-effectiveness threshold for the: (A) health system and (B) societal perspectives, based on 2,000 model simulations. The frontier identifies the vaccination strategy with the highest expected net benefit for each threshold value; of note, the optimal strategy based on expected net benefit (as indicated by the frontier) does not always correspond to the strategy with the highest probability of being cost-effective. Results are only shown for vaccination strategies with a probability of cost-effectiveness of 0.1 or greater. Productivity loss for the societal perspective was calculated using the human capital method. Note: HR = higher risk (one or more chronic medical conditions).

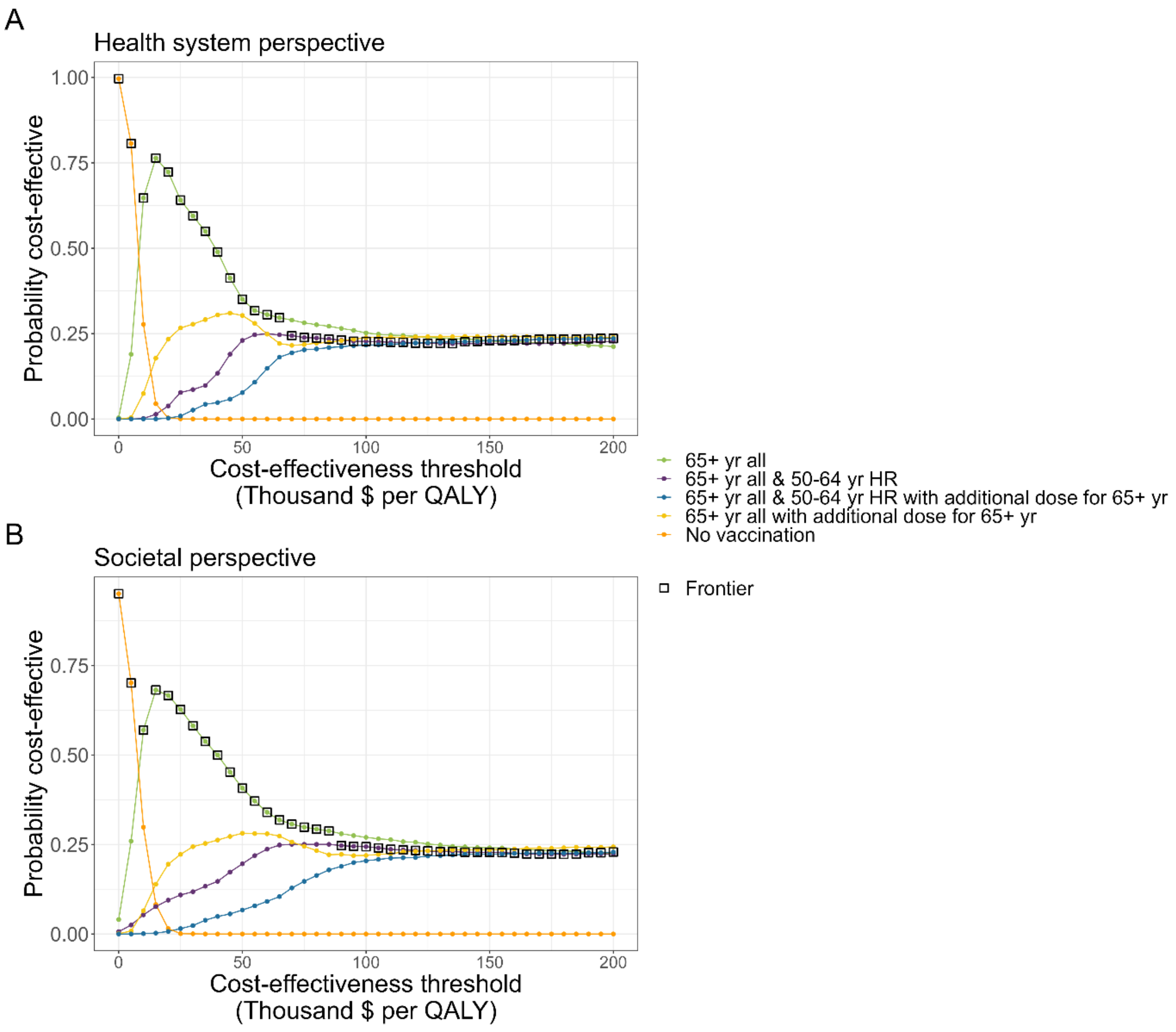

© 2025 Miranda RN et al. *JAMA Network Open*.

eFigure 5. Optimal Vaccination Strategy for a Range of Vaccine Price and Cost-Effectiveness Thresholds. The optimal strategy is the strategy with a sequential incremental cost-effectiveness ratio less than or equal to the cost-effectiveness threshold and is denoted by the colour of the bar for the indicated vaccine price per dose. The base-case analysis assumed a vaccine price of $43 per dose. For reference, dashed vertical lines indicated cost-effectiveness thresholds of $30,000, $50,000, and $70,000 per QALY. Note: HR = higher risk (one or more chronic medical conditions).

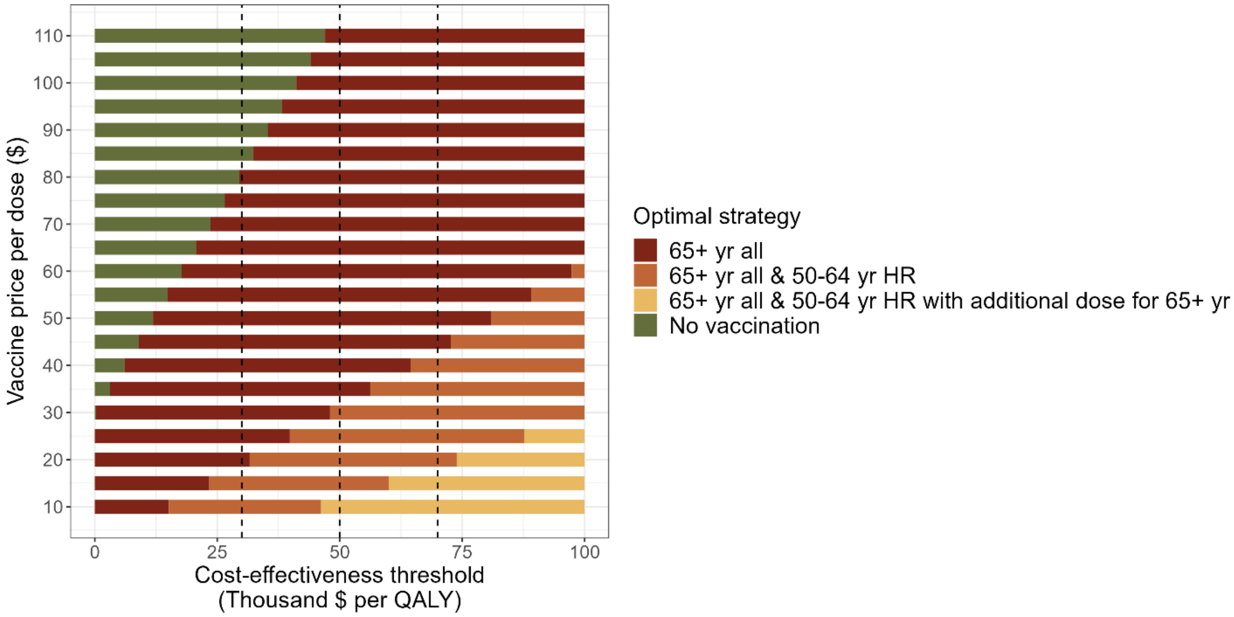

© 2025 Miranda RN et al. *JAMA Network Open*.

eTable 1. Summary of Assumptions Used in Base-Case and Scenario Analyses.

Scenario	Scenario assumptions*
Base case	• $43 per dose of vaccine (40% of CDC list price)<br>• 10% vaccine wastage<br>• VE for HR population is 0.8-fold the VE for AR population<br>• Median estimate of COVID incidence from transmission model<br>• Monthly distribution of cases estimated from 2023-2024 hospitalization data<br>• Annual dose vaccine protection starts in October/November<br>• Second dose protection starts in April/May, for strategies including a second dose for the population aged 65 and older
Higher vaccine price ($80 per dose)	• $80 per dose of vaccine (75% of CDC list price)
Higher vaccine price ($107 per dose)	• $107 per dose of vaccine (100% of CDC list price)
Higher vaccine wastage (20%)	• 20% administration-based vaccine wastage
Higher vaccine wastage (30%)	• 30% administration-based vaccine wastage
Equal VE for HR and AR groups	• VE for HR population is the same as the VE for AR population
Higher COVID-19 incidence	• COVID-19 incidence assumed to be 2-times the base-case value
Alternate seasonality (flatter curve)	• Monthly distribution of cases estimated from 2023-2024 Respiratory Virus Detection Surveillance System data
Alternate seasonality (larger winter wave)	• Monthly distribution of cases estimated from transmission model
Earlier program start	• Annual dose vaccine protection starts in August/September
Earlier program start and 4-month interval between doses	• Annual dose vaccine protection starts in August/September<br>• Second dose protection starts in December/January, for scenarios including a second dose for the population aged 65 and older

*For each scenario except the base case, any changes from the base case are indicated. All relevant base-case scenario assumptions are provided for reference.

Note: AR = average risk (no chronic medical conditions); HR = higher risk (one or more chronic medical conditions).

© 2025 Miranda RN et al. *JAMA Network Open*.

eTable 2. Health Outcomes by Vaccination Strategy, Median and 95% Credible Interval.

Strategy	Second dose for 65+ yr?	Cases per 100,000 person-years				Cases averted compared to no vaccination (%)				Number needed to vaccinate to avert one outcome			
		Outpatient	Inpatient	PCC	Death	Outpatient	Inpatient	PCC	Death	Outpatient	Inpatient	PCC	Death
No vaccination	--	8665 (8616 - 8713)	133 (127 - 140)	178 (138 - 225)	16 (14 - 18)	--	--	--	--	--	--	--	--
65+ yr all	No	8467 (8418 - 8515)	123 (117 - 130)	173 (135 - 219)	14 (12 - 16)	2.3 (2.2 - 2.4)	7.8 (6.4 - 9.4)	2.7 (2.0 - 3.4)	8.7 (5 - 12.8)	42 (40 - 44)	792 (659 - 966)	1712 (1254 - 2510)	6058 (3952 - 11374)
	Yes	8458 (8409 - 8505)	122 (116 - 129)	173 (135 - 219)	14 (12 - 16)	2.4 (2.3 - 2.5)	8.3 (6.9 - 9.8)	2.8 (2.1 - 3.5)	9.2 (5.4 - 13.7)	48 (47 - 50)	905 (761 - 1104)	1999 (1447 - 2902)	6905 (4609 - 12438)
65+ yr all & 50-64 yr HR	No	8427 (8378 - 8473)	122 (116 - 129)	172 (134 - 218)	14 (12 - 16)	2.7 (2.7 - 2.8)	8.3 (6.8 - 9.8)	3.2 (2.5 - 4)	9.0 (5.3 - 13.2)	42 (40 - 43)	902 (757 - 1102)	1727 (1266 - 2474)	6890 (4582 - 12367)
	Yes	8418 (8369 - 8465)	122 (115 - 129)	172 (134 - 218)	14 (12 - 16)	2.9 (2.8 – 3.0)	8.7 (7.2 - 10.2)	3.4 (2.6 - 4.1)	9.5 (5.5 - 14)	49 (47 - 50)	1034 (878 - 1252)	2021 (1464 - 2936)	7886 (5343 - 13658)
65+ yr all & <65 yr HR	No	8408 (8359 - 8455)	122 (116 - 129)	172 (134 - 218)	14 (12 - 16)	3.0 (2.9 - 3.1)	8.4 (6.9 - 9.9)	3.4 (2.7 - 4.2)	9.0 (5.4 - 13.2)	42 (41 - 44)	976 (822 - 1187)	1782 (1319 - 2515)	7547 (5020 - 13554)
	Yes	8399 (8350 - 8446)	122 (115 - 129)	172 (134 - 218)	14 (12 - 16)	3.1 (3.0 - 3.2)	8.8 (7.4 - 10.3)	3.6 (2.8 - 4.4)	9.6 (5.5 – 14.0)	50 (48 - 51)	1120 (949 - 1357)	2098 (1516 - 2987)	8640 (5858 - 14957)

© 2025 Miranda RN et al. *JAMA Network Open*.

50+ yr all & <50 yr HR	No	8367 (8319 - 8414)	122 (116 - 129)	171 (133 - 216)	14 (12 - 16)	3.4 (3.3 - 3.5)	8.4 (6.9 - 9.9)	4.0 (3.1 - 4.8)	9.0 (5.4 - 13.2)	43 (42 - 45)	1152 (970 - 1404)	1822 (1359 - 2551)	8916 (5932 - 16007)
	Yes	8359 (8310 - 8405)	122 (115 - 129)	171 (133 - 217)	14 (12 - 16)	3.5 (3.4 - 3.7)	8.8 (7.4 - 10.3)	4.1 (3.3 - 4.9)	9.6 (5.5 - 14)	51 (49 - 52)	1322 (1121 - 1605)	2151 (1577 - 3039)	10205 (6918 - 17683)

Note: HR = higher risk (one or more chronic medical conditions); QALY = quality-adjusted life year; ICER = incremental cost-effectiveness ratio.

© 2025 Miranda RN et al. *JAMA Network Open*.

eTable 3. Costs, QALYs, and Sequential ICERs for Base-Case Scenario by Strategy: Health System Perspective.

Strategy	Costs ($)	Effect (QALYs lost)	Incremental Costs ($)	Incremental Effect (QALYs gained)	Sequential ICER
No vaccination	90,305,263	2,889.792	--	--	--
65+ yr all annual	91,822,017	2,696.031	1,516,754	193.7606	7,828
65+ yr all & 50-64 yr HR annual	92,781,484	2,682.206	959,468	13.8254	69,399
65+ yr all biannual & 50-64 yr HR annual	94,192,769	2,671.942	1,411,284	10.2635	137,505
65+ yr all biannual & <65 yr HR annual	95,007,415	2,669.033	814,646	2.9097	279,975
65+ year all biannual, 50-64 yr all & <50 yr HR annual	96,816,201	2,665.619	1,808,786	3.4134	529,907
65+ yr all & <65 yr HR annual	93,432,115	2,679.163	--	--	Extended dominated
65+ yr all biannual	92,945,261	2,686.134	--	--	Dominated
50+ yr all & <50 yr HR annual	94,901,680	2,675.781	--	--	Dominated

Note: HR = higher risk (one or more chronic medical conditions); QALY = quality-adjusted life year; ICER = incremental cost-effectiveness ratio.

© 2025 Miranda RN et al. *JAMA Network Open*.

eTable 4. Costs, QALYs, and Sequential ICERs for Base-Case Scenario by Strategy: Societal Perspective (Human Capital Method).

Strategy	Costs ($)	Effect (QALYs lost)	Incremental Costs ($)	Incremental Effect (QALYs gained)	Sequential ICER
No vaccination	196,626,076	2,889.792	--	--	--
65+ yr all annual	198,068,598	2,696.031	1,442,522	193.7606	7,445
65+ yr all & 50-64 yr HR annual	199,270,659	2,682.206	1,202,062	13.8254	86,946
65+ yr all biannual & 50-64 yr HR annual	201,179,391	2,671.942	1,908,731	10.2635	185,972
65+ yr all biannual & <65 yr HR annual	202,351,295	2,669.033	1,171,904	2.9097	402,756
65+ year all biannual, 50-64 yr all & <50 yr HR annual	205,156,498	2,665.619	2,805,204	3.4134	821,820
65+ yr all & <65 yr HR annual	200,161,866	2,679.163	--	--	Extended dominated
65+ yr all biannual	199,471,633	2,686.134	--	--	Dominated
50+ yr all & <50 yr HR annual	202,358,450	2,675.781	--	--	Dominated

Note: HR = higher risk (one or more chronic medical conditions); QALY = quality-adjusted life year; ICER = incremental cost-effectiveness ratio.

© 2025 Miranda RN et al. *JAMA Network Open*.

eTable 5. Costs, QALYs, and Sequential ICERs for Base-Case Scenario by Strategy: Societal Perspective (Friction Cost Method).

Strategy	Costs ($)	Effect (QALYs lost)	Incremental Costs ($)	Incremental Effect (QALYs gained)	Sequential ICER
No vaccination	177,972,315	2,889.792	--	--	--
65+ yr all annual	180,085,082	2,696.031	2,112,767	193.7606	10,904
65+ yr all & 50-64 yr HR annual	181,528,768	2,682.206	1,443,686	13.8254	104,423
65+ yr all biannual & 50-64 yr HR annual	183,478,935	2,671.942	1,950,167	10.2635	190,009
65+ yr all biannual & <65 yr HR annual	184,715,092	2,669.033	1,236,157	2.9097	424,839
65+ yr all biannual & 50-64 yr all & <50 yr HR annual	187,530,329	2,665.619	2,815,237	3.4134	824,759
65+ yr all biannual	181,521,235	2,686.134	--	--	Extended dominated
65+ yr all & <65 yr HR annual	182,489,702	2,679.163	--	--	Extended dominated
50+ yr all & <50 yr HR annual	184,695,465	2,675.781	--	--	Dominated

Note: HR = higher risk (one or more chronic medical conditions); QALY = quality-adjusted life year; ICER = incremental cost-effectiveness ratio.

© 2025 Miranda RN et al. *JAMA Network Open*.

eReferences

1. Tuite AR, Simmons AE, Rudd M, et al. Respiratory syncytial virus vaccination strategies for older Canadian adults: a cost–utility analysis. *CMAJ*. 2024;196(29):E989-E1005. doi:10.1503/cmaj.240452
2. Statistics Canada. Table 17-10-0057-01. Projected population, by projection scenario, age and sex, as of July 1 (x 1,000). Accessed January 5, 2024. https://doi.org/10.25318/1710005701-eng
3. Queenan JA, Wong ST, Barber D, Morkem R, Salman A. *The prevalence of common chronic conditions seen in Canadian primary care: results from the Canadian Primary Care Sentinel Surveillance Network*. 2021. *Canadian Primary Care Sentinel Surveillance Network (CPCSSN)*.
4. Statistics Canada. Table 13-10-0777-01 Number and percentage of adults (aged 18 years and older) in the household population with underlying health conditions, by age and sex (two-year period). Accessed October 28, 2024. https://doi.org/10.25318/1310077701-eng
5. Walker S, Guan W, Freeman J, Bolker B, Flynn-Primrose D. macpan2: Fast and Flexible Compartmental Modelling. R package version 1.12.0. Accessed November 14, 2024. https://canmod.github.io/macpan2/
6. Government of Canada. COVID-19 epidemiology update. Accessed July 31, 2024. https://health-infobase.canada.ca/covid-19/current-situation.html?stat=num&measure=deaths_total&map=pt#a2
7. Prosser LA. Economic analysis of COVID-19 vaccination [slides presented at Advisory Committee on Immunization Practices (ACIP) meeting, 2024 June 24]. Accessed November 14, 2024. https://www.cdc.gov/vaccines/acip/meetings/downloads/slides-2024-06-26-28/05-COVID-Prosser-508.pdf
8. Molina M, Humphries B, Guertin JR, Feeny D, Tarride JE. Health Utilities Index Mark 3 scores for children and youth: Population norms for Canada based on cycles 5 (2016 and 2017) and 6 (2018 and 2019) of the Canadian Health Measures Survey. *Health Rep*. Feb 15 2023;34(2):29-39. doi:10.25318/82-003-x202300200003-eng
9. Yan J, Xie S, Johnson JA, et al. Canada population norms for the EQ-5D-5L. *Eur J Health Econ*. 2024;25(1):147-155. doi:10.1007/s10198-023-01570-1
10. Sander B, Mishra S, Swayze S, et al. Short-term and long-term healthcare costs attributable to diagnosed COVID-19 in Ontario; Canada: a population-based matched cohort study. *medRxiv*. 2024;doi:10.1101/2024.09.04.24313064
11. Link-Gelles R. Effectiveness of COVID-19 (2023-2024 formula) vaccines [slides presented at Advisory Committee on Immunization Practices (ACIP) meeting, 2024 June 27]. Accessed November 14, 2024. https://www.cdc.gov/acip/downloads/slides-2024-06-26-28/03-COVID-Link-Gelles-508.pdf
12. Public Health Agency of Canada. Interpretation Guide - Health Economics. Accessed November 13, 2024. https://www.canada.ca/en/public-health/services/immunization/national-advisory-committee-on-immunization-naci/methods-process/interpretation-guide-health-economics.html

© 2025 Miranda RN et al. *JAMA Network Open*.
