# Supplementary material for: Cost-Utility Analysis of COVID-19 Vaccination Strategies for Endemic SARS-CoV-2
Source: JAMA Netw Open. 2025 Jun 13;8(6):e2515534. doi: 10.1001/jamanetworkopen.2025.15534 (PMC12166483; doi:10.1001/jamanetworkopen.2025.15534)
Supplement: Supplement 2. — Data Sharing Statement [file jamanetwopen-e2515534-s002.pdf]

Data Sharing Statement

Miranda. Cost-Utility Analysis of COVID-19 Vaccination Strategies for Endemic SARS-CoV-2. *JAMA Netw Open*. Published June 13, 2025. doi:10.1001/jamanetworkopen.2025.15534

Data
Data available: No

Additional Information
Explanation for why data not available: Data used for the study are provided.
